# Assessment of Biomechanics Following Calcaneal Fracture Treatment with Internal Plate Fixation or Ilizarov External Fixation: A Retrospective, Two-Center Study

**DOI:** 10.3390/jcm14186651

**Published:** 2025-09-21

**Authors:** Igor Kowal, Marcin Pelc, Daniele Pili, Łukasz Tomczyk, Radosław Operacz, Piotr Morasiewicz

**Affiliations:** 1 Department of Orthopaedic and Trauma Surgery, Multidisciplinary Hospital in Zgorzelec, Lubańska 11-12, 59-900 Zgorzelec, Poland; ortodoc71@gmail.com; 2 Institute of Medical Sciences, University of Opole, Oleska 48, 45-052 Opole, Poland; marcin.pelc@outlook.com; 3 Orthopedic and Trauma Department, G B. Mangioni Hospital, Via L. Da Vinci 49, 23900 Lecco, Italy; info@drpiliortopedico.it; 4 Department of Food Safety and Quality Management, Poznan University of Life Sciences, Wojska Polskiego 31, 60-624 Poznań, Poland; tomczyk@up.poznan.pl; 5 Department of Orthopaedic and Trauma Surgery, Institute of Medical Sciences, University of Opole, al. Witosa 26, 45-401 Opole, Poland; r.operacz@gmail.com

**Keywords:** body load distribution, balance, calcaneal fracture, internal plate fixation, Ilizarov method

## Abstract

**Background:** There is no consensus on the best treatment method for calcaneal fractures. The topic of lower limb biomechanics following calcaneal fracture treatment with various fixation methods has not been fully explored. The aim of the study was to assess the balance and load distribution of the lower limbs in patients after various methods of stabilization of calcaneal fractures. **Methods:** In this two-center study, we retrospectively collected data from 19 patients treated with internal plate fixation at a mean age of 46 years and 27 patients treated with Ilizarov external fixation at a mean age of 50 years. Using the Zebris Medical pedobarophragmatic platform, we assessed the percentage distribution of lower limb loads and balance. **Results:** There were no significant differences in total load distribution for both the operated (*p* = 0.489) and non-operated limb (*p* = 0.46), between the Ilizarov method group and the internal plate group. In the Ilizarov fixation group, total load distribution was 46.89% on the treated limb, and 53.11% on the uninjured limb, *p* = 0.077. In the internal plate fixation group, the mean total load distribution was 41.57% in the treated limb, and 57.89% in the uninjured limb, *p* = 0.008. The median CoG (center or gravity) sway path length was 132.41 cm and 170.21 cm in the Ilizarov and internal plate group, respectively, *p* = 0.023. The median CoG sway areas were 0.84 cm^2^ and 7.57 cm^2^ in the Ilizarov method group and internal plate fixation group, respectively, *p* < 0.001. **Conclusions:** The Ilizarov method was associated with more symmetrical load distribution and improved balance performance compared to internal plate fixation. Static biomechanical parameters of calcaneal fracture treatment were better in the Ilizarov group compared to patients with internal plate fixation.

## 1. Introduction

Calcaneal fractures constitute approximately 1–2% of all fractures and 60–75% of all tarsal fractures. They most commonly occur in young and working-age adults (20–50 years old), predominantly in men [1,2]. Fractures of the calcaneus are typically a result of road traffic accidents (also those involving a motorcycle) and falls from a height [3,4]. Other risk factors include high-risk (e.g., extreme or contact) sports and—particularly in the elderly population—osteoporosis [5,6]. Geographic and socioeconomic differences in the rates of calcaneal fractures are associated with access to healthcare, road safety standards, and the level of physical activity in a given population [7].

The treatment of calcaneal fractures depends on the nature and extent of the injury. Simple nondisplaced fractures are treated with short leg cast immobilization for several weeks [8]. Intra-articular fractures, which constitute between 60% and 75% of all calcaneal fractures [1,2,3,4], are particularly challenging due to the complexity of foot anatomy and biomechanics and a high risk of complications, such as osteoarthritis, complex regional pain syndrome (CRPS), and long-term functional limitations [9,10,11,12,13,14,15,16,17]. As a result, choosing an optimal treatment strategy is essential to achieving good outcomes [3]. Displaced, intra-articular, or comminuted fractures are most typically treated surgically, either by means of open reduction and internal fixation (ORIF) with plates and screws or, less invasively, by fixation with screws or external fixation (e.g., with an Ilizarov external fixator) [1,3,4,5,7,9,10,11,12,13,14,15,16,17,18,19,20,21,22,23,24,25,26,27,28,29,30,31,32,33,34,35,36,37]. Treatment method is selected based on a number of factors, including the patient’s age and general health status, fracture class, and surgeon’s preference [13,14]. Postoperative rehabilitation is long-lasting and requires an individual approach [15]. Despite such a broad spectrum of treatment methods, there is still no consensus as to the method that can ensure the best possible functional outcomes of the injured foot [3,11,12,13,16,17,18,19,20,24,25,26,27,28,29,30,31,37,38,39].

Calcaneal fractures, particularly those involving articular surfaces, are particularly difficult to treat. These fractures significantly alter body weight distribution within the foot, causing excessive strain to other structures and increasing the risk of complications [16,17,18]. Disruptions to articular surfaces result in development of degenerative changes and limit the range of motion [19,20,21,24]. Additionally, biomechanical abnormalities of the foot often result in pain that may radiate to other parts of the body, such as the knee, the hip, or the spine [22,39].

The topic of lower limb biomechanics following calcaneal fracture treatment with various fixation methods has not been fully explored. To date, there have been studies on selected gait parameters and load distribution in the lower limbs following calcaneal fracture treatment with internal plate fixation [10,22,23,33,34,35,36]. There is limited data comparing the impact of different fixation methods on balance and weight distribution with various methods of fixation (an internal plate vs. an external Ilizarov fixator).

A thorough understanding of the complex biomechanics of the foot is essential for effective diagnostics and treatment of foot injuries and for planning effective rehabilitation programs. Advanced methods for analyzing human locomotion, for example, pedobarography, which provide a detailed assessment of balance and load distribution within the sole of the foot, help identify biomechanical abnormalities and assess treatment effectiveness [2,10,22,23,33,34,35,37,38,39,40,41]. Static pedobarography offers objective advantages in evaluating foot load distribution, including precise measurement of pressure distribution patterns, identification of areas of abnormal loading, and reliable, non-invasive assessment of plantar pressure without the influence of dynamic variables [2,10,22,23,33,34,35,37,38,39,40,41].

We posited a hypothesis that the type of calcaneal fracture fixation would affect biomechanical outcomes. The purpose of our study was to analyze biomechanical parameters in an attempt to compare two different methods of calcaneal fracture treatment. We focused on measuring plantar load distribution and assessing balance in patients who underwent calcaneal fracture treatment with either internal (plate) or external (Ilizarov apparatus) fixation.

## 2. Materials and Methods

This two-center retrospective study assessed patients treated for intra-articular calcaneal fractures by means of either an internal plate or a Polish modification of the Ilizarov method [24,25]. In one of the two study centers 19 patients were treated with plate fixation (Stryker VariAx Calcaneal Plate, Portage, WI, USA) in the years 2010–2020. In the other center, 27 patients underwent treatment with the Polish modification of the Ilizarov method in the years 2020–2022 [24,25].

Study inclusion criteria were Sanders type II, III, and IV intra-articular calcaneal fractures, no history of fractures in the affected limb, and no comorbidities in the affected limb. The follow-up period was at least 2 years after treatment completion. Other inclusion criteria were patient’s written informed consent, complete medical and radiological records. Study exclusion criteria were previous fractures of the ipsilateral foot or lower limb, other lower limb abnormalities, a follow-up period of less than 2 years, and a lack of consent. Each patient was informed of the voluntary nature of their participation in this study. The study was approved by a local ethics committee.

The surgery in all patients from both groups was performed 3 to 5 days after the injury, depending on the availability of the operating theater and the presence of an experienced orthopedist. All procedures of internal plate fixation via an extended lateral approach (ELA) in a lateral recumbent or prone position were performed by one of two experienced orthopedic surgeons. A standard extended L-shaped incision was made to protect blood vessels, nerves, and peroneal muscle tendons, and a 1.6 mm Kirschner wire was used as a retractor. Following calcaneus exposure, bone fragments were repositioned to reconstruct the articular surface and fixated with Kirschner wires and an internal plate with cortical and locking screws.

Ilizarov external fixation (ring fixation) procedures were performed under spinal anesthesia by one orthopedic surgeon. Two leg rings were secured to the tibia and fibula, a Kirschner wire was inserted into the calcaneal tuberosity. The wire was then attached to a calcaneal semiring. The semiring was then attached with connectors to the distal leg ring, and fracture was reduced under fluoroscopy. This modified configuration of the Ilizarov external fixator helped reposition bone fragments along the frontal and sagittal planes [24,25].

Patients treated with plate fixation via the ELA were fitted with a cast until the first radiological follow-up and suture removal 14 days later. The cast was then replaced with a Walker brace. The next follow-up visit was scheduled for postoperative day 6 unless there were problems with the surgical wound. Once the cast was replaced with the lower leg brace, patients were allowed to gradually increase weight bearing, initially restricted to tolerable pain levels. The treatment protocol was modified to include follow-up X-rays, included to achieve full weight bearing at three months after surgery.

Patients treated with the Ilizarov method were allowed to begin walking with elbow crutches and bear partial weight on the treated foot on postoperative day one. As pain subsided, the patients were allowed to gradually increase weight bearing, which facilitated rehabilitation. Radiological follow-up visits were scheduled every 4–6 weeks. Fracture healing progress was assessed based on radiological and clinical evidence, such as the absence of pain or the absence of pathological mobility of bone fragments. Once clinical and radiological evidence of bone union was established, the Ilizarov fixator was loosened, which facilitated full weight bearing on the treated limb. The fixator was removed if there was no secondary displacement of bone fragments and there was definitive evidence of bone union [24,25].

### 2.1. Subjects

Application of the inclusion and exclusion criteria revealed a group of 19 patients (19 males) at a mean age of 46 years (between 23 and 66 years old), whose fracture was treated with internal plate fixation and 27 patients treated with Ilizarov external fixation (1 female and 26 males), at a mean age of 50 years (from 28 to 73 years). The two patient groups did not differ significantly in terms of patient age and BMI. The internal fixation group comprised five, nine, and five patients with Sanders type II, III, and IV fractures, respectively; and the Ilizarov external fixation group comprised four, 17, and six patients with Sanders type II, III, and IV fractures, respectively.

### 2.2. Protocol of Pedobarographic Analysis

**Figure 1:**
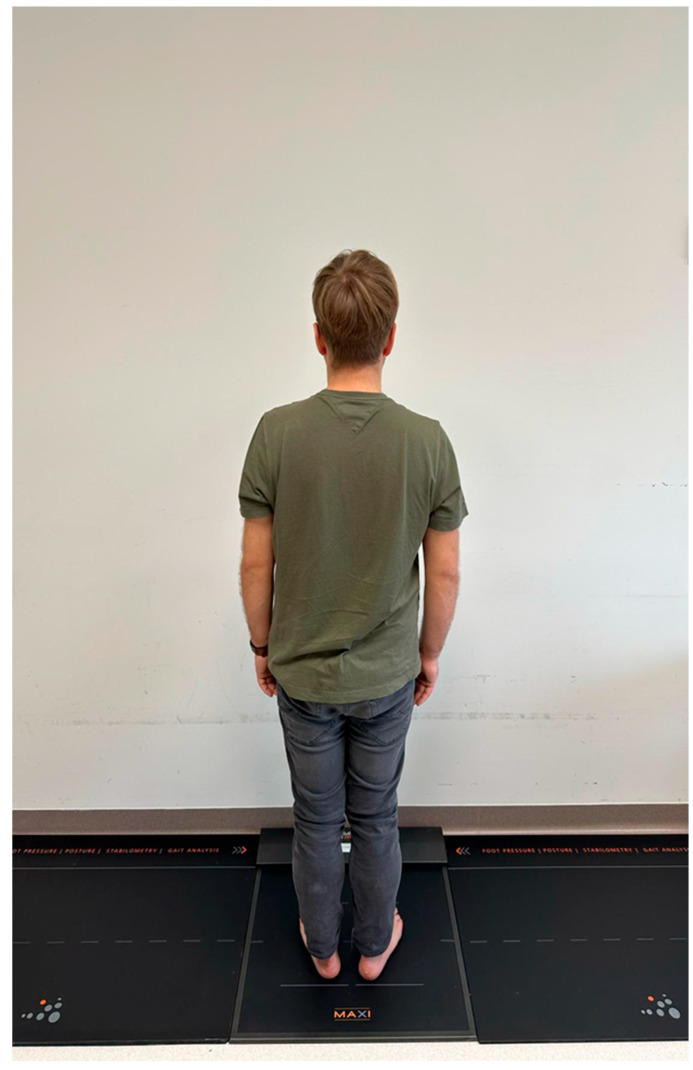
Patient on the Zebris pedobarographic platform.

Pedobarographic assessment was conducted by means of a Zebris Medical GMBH platform (320 × 470 mm) with 1504 sensors, Figure 1.

Weight distribution and balance were assessed. The data were transferred to a computer via a USB connection, and FootPrint software (version 1.2.4.9) was used to analyze static force distribution in two and three dimensions. Before measurements, each patient was informed of the purpose of the study, familiarized with the platform, and given precise instructions as to the course of the assessment. Patients could ask questions during the assessment and received corrective feedback if they deviated from its normal course. The pedobarographic platform was calibrated before each measurement. All patients underwent the 60 s assessments barefoot, with their eyes open. The examined patient would stand motionless on the platform in a neutral position, with the feet hip-width apart. Each patient underwent three measurements, and their mean value was taken for subsequent analyses.

The following parameters were recorded and analyzed (each was expressed as a percentage): total load on the treated limb [%]; total load on the untreated limb [%]; forefoot load in the treated limb [%]; forefoot load in the uninjured limb [%]; hindfoot load in the treated limb [%]; and hindfoot load in the uninjured limb [%].

Balance was assessed based on the center of gravity (CoG) sway path and area. The CoG sway path was expressed as the total distance (in centimeters) covered by the CoG during the assessment [38,39]. The other evaluated balance parameter—CoG sway area—was the area (in square centimeters) demarcated by the maximum sway of the CoG in all directions during the assessment [38,39]. The recorded data were then processed, saved, and evaluated in a subsequent statistical analysis. Balance and lower limb load distribution were compared between the patients treated with internal plate fixation and those treated with Ilizarov external fixation.

### 2.3. Statistical Analysis

Data were statistically analyzed using the Statistica 14.1 package. The Shapiro–Wilk test was used to check the normality of distribution. The t test for independent samples and the Mann–Whitney U test were used to compare quantitative variables. The chi-square test was used to determine the relationship between qualitative variables. The level of statistical significance was set at *p* < 0.05.

## 3. Results

Detailed results of lower-limb load distribution analysis have been presented in Table 1 and Table 2.

The pedobarographic measurements showed no significant differences in total load distribution on the treated limb between the Ilizarov method group and the internal plate group, Table 1. Moreover, there were no differences in total load distribution on the uninjured limb between the two groups, Table 1. We observed no significant differences between the study groups in forefoot load distribution in the treated limb, forefoot load distribution in the uninjured limb, hindfoot load distribution in the treated limb, or hindfoot load distribution in the uninjured limb, Table 1.

**Figure 2:**
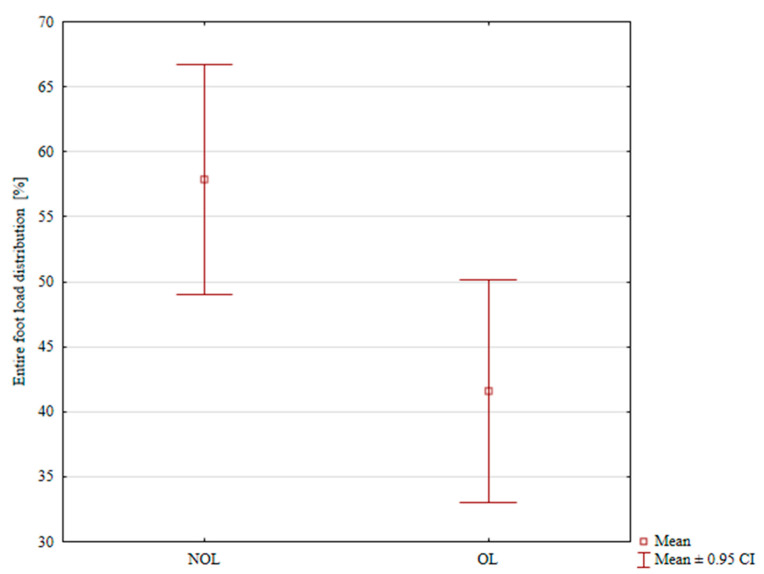
Load distribution on the treated and uninjured limb in the internal plate fixation group.

In the Ilizarov fixation group, total load distribution was 46.89% on the treated limb, and 53.11% on the uninjured limb; the difference was not statistically significant, *p* = 0.077, Table 2. In the internal plate fixation group, the mean total load distribution was 41.57% in the treated limb, and 57.89% in the uninjured limb; the difference was statistically significant, *p* = 0.008, Table 2, Figure 2.

Within the internal plate fixation group, there were no significant differences between the treated and the uninjured limb either in forefoot or hindfoot load distribution, Table 2. Similarly, pedobarographic assessments of the Ilizarov method group showed a lack of significant differences between the treated and the uninjured limb in forefoot and hindfoot load distribution, Table 2.

**Figure 3:**
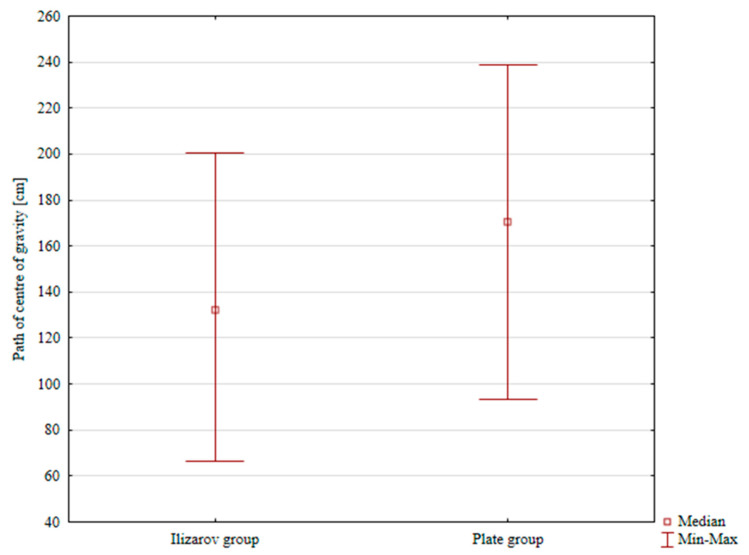
Center of gravity sway in both groups.

The median CoG sway path length was 132.41 cm and 170.21 cm in the Ilizarov and internal plate group, respectively; the difference was statistically significant, *p* = 0.0236, Table 3, Figure 3.

**Figure 4:**
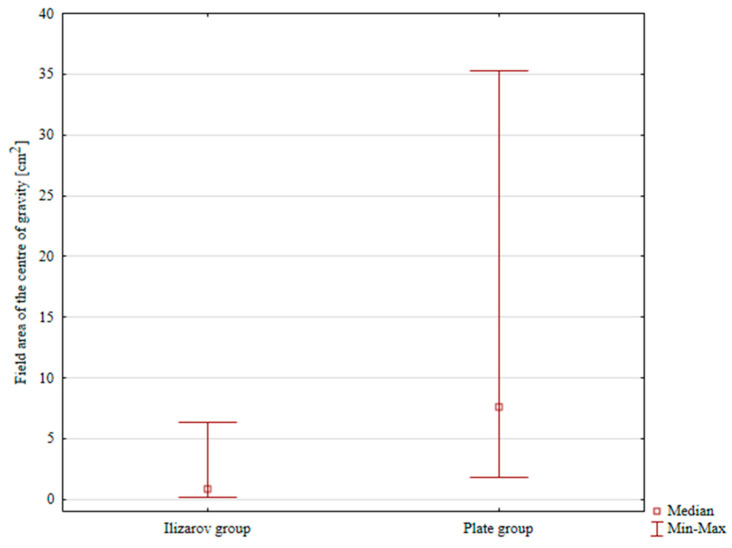
Center of gravity sway area in both groups.

The median CoG sway areas were 0.84 cm^2^ and 7.57 cm^2^ in the Ilizarov method group and internal plate fixation group, respectively; the difference was statistically significant, *p* < 0.001, Table 3, Figure 4.

## 4. Discussion

The purpose of our study was to assess and compare body weight distribution and balance in patients with calcaneal fractures treated with two different surgical approaches. Statistical analysis showed no significant differences between the study groups in total, forefoot, or hindfoot load distribution. We observed no differences in terms of load distribution between the treated and the uninjured limb in the Ilizarov fixation group. There was a significantly lower total load distribution on the treated limb than on the uninjured limb in the internal plate fixation group, whereas forefoot and hindfoot load distribution parameters in this group showed no differences between the treated and the uninjured limb. Balance parameter analysis showed significantly better outcomes in terms of both CoG sway path and CoG sway area in the Ilizarov method group than those in the internal plate fixation group. These results only partially support our research hypothesis.

Calcaneal fractures, particularly intra-articular ones, may considerably impair foot biomechanics. Articular surface damage, which is not uncommon in calcaneal fractures, may lead to osteoarthritis and chronic pain [16,19,32,39,42,43]. Biomechanical abnormalities may produce pain, swelling, limited range of motion, and abnormal load distribution over the sole of the foot [22,33,39]. Consequently, affected patients experience difficulties while walking, running, and performing other physical activities, which adversely affects their quality of life. Complications like CRPS pose additional problems during the course of treatment and worsen the prognosis [33]. Calcaneal fractures often result in long-term inability to resume work, which leads to loss of income and potential poverty, particularly for blue-collar workers or those whose occupation requires physical activity. Time to resuming work varies and depends on a number of factors, including fracture type and the effectiveness of rehabilitation [1,2,3,8,9,10,11,13,24,25,26,29,30,31,39,42,43]. Asymmetrical load distribution and abnormal balance parameters may be due to pain, limited range of motion, and muscle atrophy [33,35,38,39]. Chronic pain, limited range of motion, and foot dysfunction following calcaneal fracture greatly lower the quality of life. These symptoms also adversely affect physical activity, recreation, work, social relations, and general wellbeing, which consequently lowers the patient’s quality of life and leads to mental problems [7,8,9,10,11,12,13,21,22,23,24,25,26,29,31,32,33,34]. Systematic review and meta-analysis showed that minimally invasive approaches compared to extensive lateral approaches in the treatment of calcaneal fractures allow for similar functional results and anatomical reduction [43]. The above study may encourage less invasive treatment of calcaneal fractures due to the potential benefits of a small surgical approach—smaller scar, lower risk of complications [43].

In one of their reports, Hetsroni et al. advocated for ORIF in high-grade calcaneal fractures [34]. Although this treatment approach helped effectively restore normal kinematics of the ankle joint and produced satisfactory functional outcomes, it did not completely restore normal subtalar joint mobility [34]. Despite successful surgical reconstruction, mobility of the subtalar joint remained significantly limited up to two years after the procedure in comparison with that in an uninjured control group. This limitation was manifested in limited range of motion, and delayed time to maximum foot inversion. These differences were not fully accounted for by the differences in gait speed between the two groups. Kinematic analysis showed a relative bilateral symmetry of the treated and uninjured limbs, which indicates an adequately restored gait function. The authors of that study suggest that persistent limitations in subtalar range of motion may be due to such factors as cartilage damage, ligament damage, or other soft tissue injuries associated with this complex fracture pattern [34].

Jandova et al. compared plantar load distribution during gait following two different surgical procedures for calcaneal fracture treatment (i.e., the ELA and the less invasive sinus tarsi approach [STA]) [33]. Differences in load distribution between the treated foot and the uninjured foot persisted for 6 months after either procedure. Patients bore less weight on the treated foot, with a tendency to shift more weight onto the forefoot. The STA produced better outcomes. In the STA group, the differences between the treated and uninjured limb in load distribution and timing parameters were minimal. The values of dynamic and temporal variables for the treated limb were nearly identical to those in the uninjured limb [33]. The ELA produced greater differences in a dynamic assessment [33]. In the ELA group stance phase duration and total load were significantly lower in the treated foot. The stance phase in both groups was shorter in the treated foot, with the difference reaching statistical significance only in the STA group. The heel strike time was longer in the treated foot. The maximum load was higher in both groups in the uninjured foot, with the difference statistically significant in the ELA group. The heel load in both groups was significantly higher in the uninjured foot. The total load in both groups was lower in the treated foot; the difference was significant in the ELA group [33]. The authors concluded that the less invasive STA may be more effective than ELA in the management of displaced calcaneal fractures, leading to more rapid restoration of gait symmetry [33].

Brand et al. also compared functional outcomes in patients treated with the ELA versus the STA [10]. Their retrospective study was conducted in 56 patients with unilateral intra-articular calcaneal fractures; 26 and 30 patients were treated with the ELA and STA, respectively [10]. Significant improvement in functional and biomechanical outcomes was observed in both study groups between month 3 and month 6 of follow-up; ankle range of motion and foot motion improved by 34% and 26%, respectively; maximum ankle moment improved by 34% [10]. Vertical ground reaction force increased by 8%. That study showed both ELA and STA to be effective in calcaneal fracture treatment, since they ensure comparable improvement in foot function over the first six months after the procedure. The less invasive STA seems to be as effective as the conventional ELA, which may be of significance to patients. The less invasive STA ensures adequate restoration of dynamic foot function and may be a good alternative to the more commonly used ELA [10].

In another calcaneal-fracture study three years later, Hetsroni et al. demonstrated that patients with complex calcaneal fractures following ORIF exhibited persistent asymmetry of plantar load despite Bohler’s angle improvement [22]. These asymmetries were characterized by a shorter stance phase, delayed peak loading time under the first and second digit, transfer of plantar load onto the lateral part of the midfoot, and load reduction on digits III–V [22]. That study showed that patients with complex calcaneal fractures tended to have persistent abnormalities in plantar load distribution even after a long time following the ORIF procedure. These anomalies correlated with clinical outcomes, which suggests the need for modifying the treatment strategy to improve load distribution and, at the same time, improve foot function [22].

Morasiewicz et al. used a pedobarographic platform to assess 21 patients after ankle joint arthrodesis with the Ilizarov method [38]. Those authors observed a symmetrical load distribution in the lower limbs after treatment. The mean CoG sway path length was 137.9 cm, and the mean CoG sway area was 7.41 cm^2^ [38]. Pelc et al. retrospectively evaluated a group of patients with calcaneal fractures treated with a modified Ilizarov method [39]. Load distribution analysis showed no significant differences either between the treated and uninjured limb in the patient group or between the nondominant and dominant limb in the control group [39]. Calcaneal fracture treatment with the Ilizarov method normalized body weight distribution over the lower limbs, yielding outcomes comparable with those in the control group. CoG path sway was significantly greater in the group of patients treated with the Ilizarov method than in the control group, which indicates an incomplete normalization of balance parameters [39]. Genc et al. assessed 28 patients with intra-articular calcaneal fractures treated surgically [35]. Dynamic pedobarographic analysis conducted after a mean of 22.25 months after the procedure showed that, despite only a slight difference in the arch index (AI) between the treated and uninjured foot (29.73% vs. 28.94%, *p* = 0.078), there were significant changes in plantar pressure distribution [35]. The treated feet showed a significant decrease in the maximum pressure under the second, third, fourth, and fifth metatarsals and the medial part of the heel (*p* < 0.05). Heel pressure shifted laterally (*p* = 0.029). Although total stance phase duration did not differ significantly between the treated and uninjured foot (1749.1 ms vs. 1401.3 ms, *p* = 0.236), the contact phase of the third and fourth metatarsals was shorter in the treated foot (*p* < 0.05). There were no significant differences in other evaluated areas (*p* > 0.05) [35]. Kuzminas et al. analyzed the relationship between ankle joint instability and plantar pressure distribution [40]. The study was conducted in 16 young, physically active individuals (10 males and 6 females) with ankle instability. Plantar pressure measurements during standing, walking, and running were conducted with a platform with an inbuilt sensor system. Individuals with ankle instability exhibited asymmetrical plantar pressure distribution [40]. The pressure during standing, walking, and running was higher on the lateral side of the sole and lower on the medial side. The greatest differences in pressure distribution were observed during running, particularly in the forefoot [40]. Individuals with ankle instability exhibited a greater internal rotation of the treated foot (a difference of 4.7 degrees) than of the uninjured foot. Changes in pressure distribution were associated with changes in the CoG path, which shifted towards the lateral part of the foot in patients with ankle instability [40]. The study showed that ankle instability affects plantar pressure distribution, leading to increased load on the lateral side of the sole and a decreased load on the medial side of the sole. These changes in pressure distribution may cause the development of pain and other symptoms association with ankle instability [40]. The authors emphasized the need for further studies to better understand this association and develop effective treatment methods [40]. Our study showed symmetrical load distribution between the treated and uninjured lower limb in the Ilizarov fixation group, which may indicate that ankle instability is normalized after this type of treatment. Another study focused on the effect of Chopart joint (transverse tarsal joint) injuries on gait kinematics and pressure distribution [41]. The study included 14 patients with isolated Chopart joint injury. Three-dimensional gait analysis, electromyography (EMG), and pedobarographic analysis were performed [41]. Gait analysis showed a significantly increased internal rotation and decreased external rotation at the hip joint on the injured side, as well as decreased adduction and range of motion at the ipsilateral ankle joint [41]. On the uninjured side, there was a significantly increased forefoot loading [41]. While standing, patients bore considerably more weight on the healthy side [41]. Patients with isolated Chopart joint injury showed significant gait abnormalities and pedobarography results at long-term follow-up [41].

We know that both groups underwent surgery at different times, but due to the rarity of calcaneal fractures, it was difficult to match patients treated with different methods with more similar follow-up periods. In both patient groups, the follow-up period was at least two years after the end of treatment. A follow-up period exceeding two years after the end of treatment is considered sufficient to achieve final functional status and achieve final muscle strength and joint mobility after treatment. In longer follow-up (over two years), parameters that may influence biomechanical outcomes (pain, joint mobility, muscle strength) no longer change significantly.

Reports indicate that calcaneal fractures constitute severe injuries, and both static and dynamic lower-limb parameters often remain abnormal after calcaneal fracture treatment with internal fixation [10,22,23,32,33,34,35,36]. Our study also showed worse biomechanical parameters following calcaneal fracture treatment with internal plate fixation than following treatment with the Ilizarov method. We observed better balance results in the Ilizarov method group than in the internal plate fixation group. Balance assessment results in our study were consistent with those reported by others [38,39], which may indicate measurement repeatability. Total load distribution on the treated and uninjured limb was comparable in patients treated with the Ilizarov method, but showed as shift in loading onto the uninjured limb in the ORIF group. The better biomechanical outcomes in the Ilizarov group may result from the earlier initiation of walking with weight bearing on the treated limb, which may have prevented muscle atrophy and diminished balance problems. Conversely, ORIF with a plate was associated with a large surgical access incision, posing potential injury to muscles, ligaments, and other soft tissues, and left a postoperative scar. The patients treated with the Ilizarov method underwent closed reduction, with no incision to expose the calcaneus, which may have prevented potential adverse effects on the assessed balance parameters. The worse results in the internal fixation group could have been influenced by postoperative immobilization in a cast and scars. The patients with calcaneal fractures treated with internal fixation require longer and more intensive rehabilitation and a longer follow-up.

The limitations of our study include a relatively small sample size. This was due to the to a low incidence of calcaneal fractures and the use of highly restrictive study inclusion and exclusion criteria. Authors of other reports on calcaneal fracture treatment assessed patient groups of similar or smaller size [9,10,22,23,24,25,33,34,35,36,38,39]. Another limitation of the study may also be a lack of pre-injury pedobarographic and functional parameters (in other words a retrospective nature of the study); however, this would have been impossible due to the fact that the experimental group comprised injuries (calcaneal fractures). We are aware that a weakness of our study is the slightly different observation periods for the two patient groups. Unfortunately, due to the low incidence of calcaneal fractures, collecting an adequate number of patients for analysis required extending the time frame. We are also aware that two different rehabilitation protocols may have had some influence on the biomechanical outcomes. Determining the extent to which they may affect foot biomechanics after calcaneal fracture requires further evaluation. One of the strengths of our study is a comparative analysis of two methods of surgical treatment for calcaneal fractures. Moreover, this was a two-center study, evaluating populations of similar size, with a comparable mean patient age, follow-up period, and sex distribution. Another strength of our study was a rehabilitation protocol and a follow-up visit schedule that were similar in both centers and in both study groups; as well as the fact that all procedures were performed by a total of three orthopedic surgeons (two performing internal plate fixation and one performing Ilizarov external fixation). In the future, we are planning to conduct a similar study in a larger population and analyze functional parameters and the gait of those patients.

Although significant differences in load distribution and balance were found between the two patient groups, these results should be interpreted with caution given the study’s clear limitations (unequal follow-up duration, lack of clinical–functional measures, and absence of defined cut-offs for clinically relevant differences in limb support). The choice of treatment should ultimately be guided by the surgeon’s experience with the respective techniques. The use of the Ilizarov method in calcaneal fractures is particularly beneficial in patients with multiple injuries, poor condition of soft tissues, edema and in the case of open fractures.

## 5. Conclusions

The Ilizarov method was associated with more symmetrical load distribution and superior balance performance compared to internal plate fixation.

Static biomechanical parameters of calcaneal fracture treatment were better in the Ilizarov group compared to patients with internal plate fixation.

The study results should be interpreted with caution due to the limitations of the study (unequal follow-up duration, lack of clinical–functional measures), and the final choice of treatment method depends on the surgeon’s experience with the respective techniques.

## Figures and Tables

**Table 1:** Body weight distribution in the Ilizarov group and plate group.

Analyzed Variable	Ilizarov Group	Plate Group	*p*
	Mean ± Standard Deviation		
Entire foot load distribution NOL [%]	53.11 ± 7.23	57.89 ± 18.35	0.46
Forefoot load distribution NOL [%]	47.33 ± 8.58	40.57 ± 16.52	0.261
Hindfoot load distribution NOL [%]	52.66 ± 8.58	59.42 ± 16.52	0.261
Entire foot load distribution OL [%]	46.89 ± 8.67	41.57 ± 17.83	0.489
Forefoot load distribution OL [%]	42.33 ± 9.48	35.94 ± 17.74	0.323
Hindfoot load distribution OL [%]	57.66 ± 9.48	64.05 ± 17.74	0.323

OL—operated limb; NOL—non-operated limb; *p*—*p*-value for the *t*-test.

**Table 2:** Body weight distribution for patients after treatment between OL and NOL.

Loads on Limb	Ilizarov Group	Plate Group
	Mean ± Standard Deviation	
entire foot load distribution NOL [%]	53.11 ± 7.23	57.89 ± 18.35
entire foot load distribution OL [%]	46.89 ± 8.67	41.57 ± 17.83
*p*	0.077	0.008
Forefoot load distribution NOL [%]	47.33 ± 8.58	40.57 ± 16.52
Forefoot load distribution OL [%]	42.33 ± 9.48	35.94 ± 17.74
*p*	0.258	0.41
Hindfoot load distribution NOL [%]	52.66 ± 8.58	59.42 ± 16.527
Hindfoot load distribution OL [%]	57.66 ± 9.48	64.05 ± 17.74
*p*	0.258	0.41

OL—operated limb; NOL—non-operated limb; *p*—*p*-value for the *t*-test.

**Table 3:** Balance in the Ilizarov group and plate group.

Analyzed Variable		Ilizarov Group	Plate Group	Z	*p*
	Value				
Path of center of gravity [mm]	Q1	107.735	156.74	−2.262	0.023
	Median	132.416	170.21		
	Q3	148.354	201.55		
Field area of the center of gravity [mm^2^]	Q1	0.608	4.5	−3.787	*p* < 0.001
	Median	0.849	7.57		
	Q3	1.806	10.04		

Z—standardized value of the Mann–Whitney test; *p*-value for the Mann–Whitney U test; Q1, Q3—1st and 3rd quartile.

## Data Availability

The data presented in this study are available on request from the corresponding author.
